# A Comparative Analysis of the Efficacy and Safety of Nimesulide/Paracetamol Fixed-Dose Combination With Other NSAIDs in Acute Pain Management: A Randomized, Prospective, Multicenter, Active-Controlled Study (the SAFE-2 Study)

**DOI:** 10.7759/cureus.58859

**Published:** 2024-04-23

**Authors:** Sandip Patil, Naushad Nadaf, Sabyasachi Gupta, Pratik Barai, Swati Makhija, Prateek Lodha, Chintan Patel, Ajitkumar A Gondane, Dattatray Pawar, Akhilesh Sharma

**Affiliations:** 1 Orthopedics, Patil Orthopedic Hospital, Karad, IND; 2 Dentistry, Shaheen Naushad Nadaf Dental Clinic, Solapur, IND; 3 Medicine, Chirayu Medical College, Bhopal, IND; 4 Medicine, Arogyam Clinic, Nagpur, IND; 5 Dentistry, Makhija Dental Clinic, Ahmedabad, IND; 6 Orthopedics, Zydus Multispeciality Hospital, Ahmedabad, IND; 7 Medicine, Aatman Hospital, Ahmedabad, IND; 8 Medical Affairs, Alkem Laboratories Ltd., Mumbai, IND

**Keywords:** post marketing study, paracetamol, gastroduodenal ulcer, nimesulide, ketorolac, fixed dose combination, diclofenac, aceclofenac

## Abstract

Objective

In this study, we aimed to compare the efficacy and safety of the fixed-dose combination (FDC) of nimesulide (100 mg) + paracetamol (325 mg) [NP], ketorolac (10 mg) [Kt] alone, diclofenac (50 mg) + paracetamol (325 mg) [DP], and aceclofenac (100 mg) + paracetamol (325 mg) [AP] in patients with acute painful conditions.

Methods

This was a randomized, prospective, open-label, multicentre, active-controlled study involving patients aged ≥18 years, with acute painful conditions like low back pain, acute musculoskeletal disorders, and trauma such as tendinitis, tenosynovitis, bursitis, sprains and strains, migraine, dental pain, painful dental procedures, and post-surgical pain. Reduction in pain intensity and liver, renal, gastrointestinal, and cardiovascular safety were assessed on days seven and 14.

Results

A total of 600 patients were randomized into NP, Kt, DP, and AP groups in a 1:1:1:1 ratio. NP, DP, and AP were administered twice a day while Kt was given three times a day. The reduction of pain as measured by the numerical rating scale (NRS) scores at the end of day seven was 3.75 ± 1.58 in the NP group, 2.96 ± 1.18 in the Kt group, 3.42 ± 1.42 in the DP group, and 3.47 ± 1.30 in the AP group. The pain reduction in the NP group was significantly greater (p<0.001) as compared to the Kt group and non-inferior to the DP and AP groups on days seven and 14. Non-inferiority was concluded between the NP, DP, and AP groups as the lower limit of 95% CI of the difference in the change of pain intensity on both days seven and 14 was above the predefined margin of -1.0. All the drugs were well tolerated, but a significantly greater number of adverse events were reported in the DP group (32) as compared to the NP group (14) (p<0.05). The most common adverse events reported during the study were nausea, gastritis, and abdominal pain in all four groups. There was no significant alteration in liver and renal function tests except a rise in serum creatinine in the DP group.

Conclusions

The FDC of nimesulide with paracetamol is superior to ketorolac and non-inferior to the FDC of diclofenac with paracetamol and aceclofenac with paracetamol in the management of pain in patients with acute painful conditions. The tolerability profile of the FDC of nimesulide with paracetamol is similar to that of ketorolac but better than diclofenac with paracetamol and aceclofenac with paracetamol combinations.

## Introduction

Pain is defined as an unpleasant sensory and emotional experience due to a true or potential tissue injury [1]. The expression and severity of pain varies significantly among individuals. Pain is affected by various factors like racial, cultural, and environmental factors, past experiences, and philosophical and mental states [1]. It is one of the leading causes of primary care consultations. Acute pain can be a warning sign of a disease and usually resolves within the normal expected healing period. However, untreated or unrelieved acute pain can turn into chronic pain and remain persistent even after the initial injury has healed.

Acute and chronic pain management requires pharmacological treatment. Nonsteroidal anti-inflammatory drugs (NSAIDs) are among the most commonly used analgesic agents for the treatment of acute pain. NSAIDs are chemically different groups of drugs with the same or less analgesic, antipyretic, and anti-inflammatory activity. Opioid receptor-independent cyclooxygenase (COX) inhibition has several advantages but is also associated with some side effects, which can be dose-dependent. At therapeutic doses, it can cause gastrointestinal ulcers, bleeding, and cerebrovascular disorders, while at high doses, it may cause damage to the kidney, liver, and heart [2-4].

Nimesulide is a preferential COX-2 inhibitor with potent anti-inflammatory, analgesic, and antipyretic activities, and it can be useful in many painful conditions. It leads to rapid and sustained control of pain and inflammation. It also has a good safety profile as it causes fewer gastrointestinal side effects as compared to other NSAIDs [5]. However, rare and unpredictable hepatic injury has been reported due to nimesulide therapy. The European Medicines Agency’s Committee for Medicinal Products for Human Use (CHMP) performed a full assessment of the benefits and risks of nimesulide in January 2010. In 2012, the CHMP concluded that the benefits of systemic nimesulide-containing medicines continue to outweigh their risks in the treatment of patients with acute pain [5]. Moreover, the duration of treatment was relatively small (15 consecutive days only). Liver damage is a rare side effect already associated with NSAIDs; recent pharmaco-epidemiological studies have shown that safety concerns about nimesulide are no higher than with other NSAIDs and that the risk/benefit profile for hepatic and kidney defects is comparable with other drugs of its class [6-8].

Ketorolac is indicated for moderate to severe acute pain including acute musculoskeletal trauma pain, post-partum uterine cramping pain, dental-related pain, and post-surgical pain. It is administered either as tablets or as an intramuscular injection for a shorter duration. The dose for oral ketorolac is 10 mg every four to six hours, not exceeding 40 mg per day [09].

Diclofenac is an NSAID that acts by inhibiting the cyclooxygenase-1 (COX-1) and cyclooxygenase-2 (COX-2). It inhibits the synthesis of inflammatory mediators like prostaglandin-E2 (PGE2), prostacyclins, and thromboxanes and also inhibits the nociceptive response. Diclofenac is indicated for the treatment of acute and chronic pain, including pain associated with ankylosing spondylitis, osteoarthritis, and rheumatoid arthritis. It is also used in migraine, myalgia, biliary colic, corneal abrasion, fever, gout, and post-episiotomy pain [10].

Aceclofenac is a drug belonging to the class of NSAIDs and is derived from phenylacetic acid; it exhibits notable anti-inflammatory and analgesic properties. This medication acts as a potent inhibitor of COX, an enzyme crucial for the synthesis of prostaglandins and thromboxanes. Notably, aceclofenac demonstrates high selectivity for the COX-2 isoform over COX-1. Originally approved in the European Union in 1990, aceclofenac has gained approval for use in several countries worldwide. While specific indications may vary across regions, it is commonly employed for treating inflammatory and painful conditions. Aceclofenac is generally well-tolerated, with dizziness, gastrointestinal disorders, and elevated liver enzymes being the most commonly reported side effects. Current evidence suggests that aceclofenac may offer better gastrointestinal tolerability when compared to other NSAIDs [11].

Various NSAIDs and their combinations with paracetamol have been approved in India and are being used for the management of many painful conditions. All these NSAIDs have some differences in their pharmacodynamics and pharmacokinetic properties, resulting in an overall impact on the efficacy and safety of the products. Data are scarce in the literature about the efficacy and safety of fixed-dose combination (FDC) of nimesulide and paracetamol. To date, only a few studies with small sample sizes and nonrandomized studies have been published to assess the efficacy and safety of nimesulide + paracetamol among the Indian population. In light of this, the current post-marketing study is being undertaken to evaluate and compare the safety and efficacy of the four commonly used combinations of NSAIDs, i.e. nimesulide (100 mg) + paracetamol (325 mg) [NP], ketorolac (10 mg) [Kt] alone, diclofenac (50 mg) + paracetamol (325 mg) [DP], and aceclofenac (100 mg) + paracetamol (325 mg) [AP] in patients with acute painful conditions such as low back pain, acute musculoskeletal disorders and trauma such as tendinitis, tenosynovitis, bursitis, sprains and strains, migraine, dental pain, painful dental procedures, and post-surgical pain.

## Materials and methods

Study design, setting, and ethical approval

This was a randomized, prospective, open-label, multicentre, active-controlled, comparative study conducted by 14 investigators in different geographical areas across India. It involved orthopedic surgeons, physicians, dentists, and general surgeons, and was performed in compliance with the ethical principles of the Declaration of Helsinki, Indian Good Clinical Practice Guidelines, and Indian Council of Medical Research (ICMR) guidelines for Biomedical Research on Human Subjects (2017). The study obtained approval from the Institutional Ethics Committees at each of the participating study centers. Written informed consent was obtained from all patients before the initiation of any study-related activity. The study was registered with the Clinical Trials Registry of India (www.ctri.nic.in; CTRI/2023/09/057622).

Patients

Patients of both genders aged ≥18 years, with acute painful conditions like low back pain, acute musculoskeletal disorders, and trauma such as tendinitis, tenosynovitis, bursitis, sprains and strains, migraine, dental pain, painful dental procedures, and post-surgical pain were included the study. The exclusion criteria were as follows: patients with a past history of hypersensitivity to any of the study drugs, those who had used NSAIDs or any other analgesic in last 24 hours, had history of GI bleeding, had clinically significant uncontrolled cardiovascular disease, had hepatic dysfunction (serum transaminases ≥3 x upper limit of normal) or renal dysfunction (serum creatinine ≥ 2.5mg/dl) at screening, pregnant and lactating mothers and women of childbearing potential not taking adequate contraceptive measures, and those prescribed/required any other medication known to interact with the study medications.

Study procedures and drugs

A total of 600 patients with acute painful conditions like low back pain, acute musculoskeletal disorders, and trauma such as tendinitis, tenosynovitis, bursitis, sprains and strains, migraine, dental pain, painful dental procedures, and post-surgical pain were enrolled in this post-marketing study. After signing the informed consent form, the subjects were screened to assess eligibility according to the study selection criteria. Upon fulfillment of the selection criteria, subjects were randomized in a 1:1:1:1 ratio and randomly prescribed either of the four study drugs, i.e., FDC of nimesulide (100 mg) + paracetamol (325 mg) [NP] or ketorolac (10 mg) [Kt] or diclofenac (50 mg) + paracetamol (325 mg) [DP], or aceclofenac (100 mg) + paracetamol (325 mg) [AP] tablets for the management of their painful conditions as per investigators' discretion based on routine clinical practice. FDC of nimesulide/diclofenac/aceclofenac + paracetamol was prescribed twice a day (maximum two tablets in 24 hours) for a maximum duration of 10 days while ketorolac was prescribed three times a day (maximum three tablets in 24 hours) for a maximum duration of seven days. There was no provision for any rescue medications. Patients not showing improvement with study medications or those whose conditions worsened were given standard treatment as per investigator’s discretion.

Patients were screened during visit 1 based on age, gender, height, weight, physical examination, past medical history, history of GI bleeding, melena, and dyspepsia-related medication history, Electrocardiogram (ECG) and laboratory investigations [hematology: hemoglobin (Hb)/complete blood count (CBC); biochemistry: alanine aminotransferase (ALT), aspartate aminotransferase (AST), serum bilirubin, blood urea nitrogen (BUN), serum creatinine]. Pain intensity was recorded on a 10-point numerical rating scale (NRS). The patients were followed up on an outpatient basis on day seven (visit 2) and day 14 (visit 3). During each clinic visit, pain intensity was recorded to evaluate the efficacy, and ECG and laboratory investigations (hematology: Hb/CBC; biochemistry: ALT, AST, serum bilirubin, BUN, serum creatinine) were done to evaluate the safety of the study drugs.

Efficacy and safety assessments

The efficacy endpoint was the reduction of pain as measured by NRS at the end of day seven and day 14 as compared to baseline among all four groups. Safety endpoints were as follows: change in liver function tests/parameters (AST, ALT, serum bilirubin), renal function test (serum creatinine and blood urea nitrogen), and ECG from baseline to day seven and day 14 in the four groups. An increase in AST and ALT of more than 1.5 times the upper limit of normal and an increase in serum bilirubin, creatinine, and blood urea above the upper limit of normal were recorded as adverse events during the study. Other treatment-emergent serious and non-serious adverse events (TEAEs) were also reported during the two-week follow-up in the four groups.

Statistical analyses

Data were analyzed using GraphPad Prism Version 10.1.0 (316) and sample size was calculated using nMaster software. A sample size of 600 subjects was calculated for three groups in the study. A sample size of 150 subjects in each group would give 80% power to detect one adverse event known to occur with a frequency of 1:100. This sample size was also deemed sufficient to show non-inferiority of the treatment medications, considering a one-sided alpha of 2.5%, power of 90%, a non-inferiority margin of 1 point on NRS [12], standard deviation (SD) of 2.5, and a dropout rate of 10%. Data were presented as mean ± SD/SE or number (percentage). Descriptive statistics were used for different variables at baseline. Repeated measures ANOVA with Dunnet’s Multiple Comparisons Test was used for the comparison within-group and one-way ANOVA with Bonferroni's multiple comparisons test was used for between-group comparisons. A p-value <0.05 was considered statistically significant. Modified intention-to-treat (mITT) analysis with Last observation Carry Forward (LOCF) was used for this study. The LOCF method has been used to impute missing data in patients who were lost to follow-up or dropouts.

## Results

**Figure 1:**
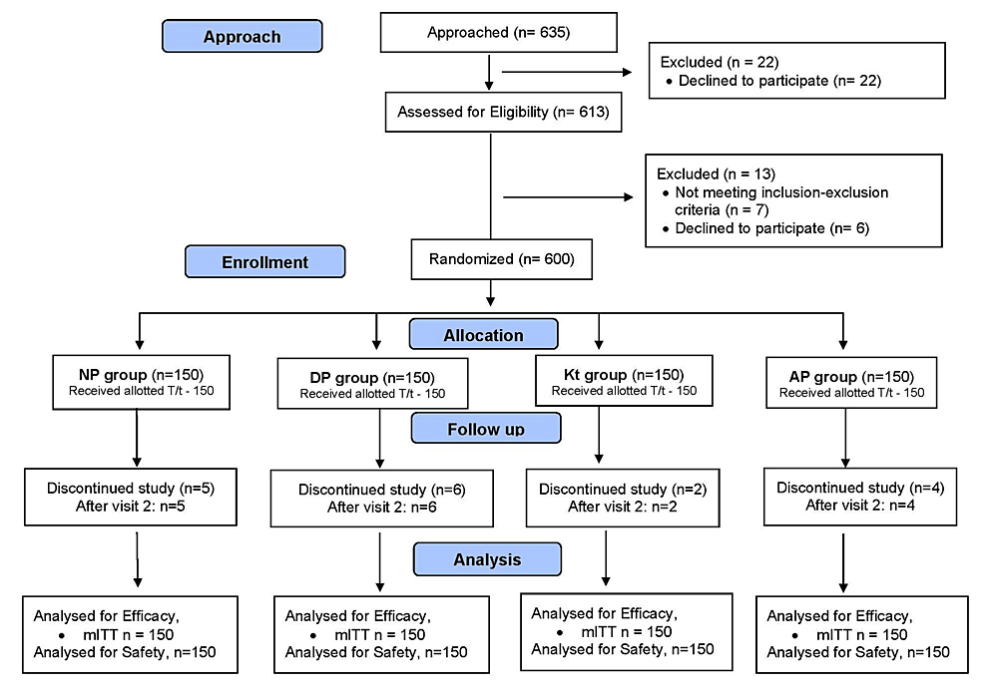
Flow chart depicting the selection of patients NP: nimesulide (100 mg) + paracetamol (325 mg) tablets; DP: diclofenac (50 mg) + paracetamol (325 mg) tablets; Kt: ketorolac (10 mg); AP: aceclofenac (100 mg) + paracetamol (325 mg) tablets; mITT: modified intention-to-treat

A total of 600 patients were enrolled in this prospective, open-label, multicentre, active-controlled, comparative, post-marketing study. A flow chart depicting the selection of patients in the study is presented in Figure 1.

The demographic details of patients enrolled in the study and the distribution of patients according to indication are shown in Table 1.

**Table 1:** Demographic characteristics of enrolled subjects SD: standard deviation; CI: confidence interval; NP: nimesulide (100 mg) + paracetamol (325 mg) tablets; DP: diclofenac (50 mg) + paracetamol (325 mg) tablets; Kt: ketorolac (10 mg); AP: aceclofenac (100 mg) + paracetamol (325 mg) tablets

Parameter	NP	DP	Kt	AP
No. of subjects enrolled	150	150	150	150
Age, years, mean ± SD (95% CI)	38.6 ± 9.86 (37.0 to 40.2)	36.8 ± 10.6 (35.1 to 38.6)	37.9± 10.3 (36.2 to 39.6)	38.2 ± 10.9 (36.4 to 39.9)
Male, n (%)	76 (50.7%)	67 (44.7%)	67 (44.7%)	70 (46.7%)
Female, n (%)	74 (49.3%)	83 (55.3%)	83 (55.3%)	80 (53.3%)
Weight, Kg, mean ± SD (95% CI)	62.5 ± 8.38 (61.1 to 63.9)	59.9 ± 6.92 (58.8 to 61.0)	61.3 ± 7.55 (60.1 to 62.5)	61.5 ± 8.16 (60.2 to 62.9)
Height, cm, mean ± SD (95% CI)	161 ± 6.14 (160 to 162)	161 ± 6.48 (160 to 162)	161 ± 6.57 (160 to 162)	161 ± 6.39 (160 to 162)
Indication, n (%)				
Low back pain	23 (15.3%)	25 (16.7%)	28 (18.7%)	27 (18%)
Dental procedure (tooth extraction)	22 (14.7%)	24 (16.0%)	22 (14.7%)	22 (14.7%)
Post-surgical	26 (17.3%)	16 (10.7%)	25 (16.7%)	22 (14.7%)
Myalgia	17 (11.3%)	20 (13.3%)	17 (11.3%)	17 (11.3%)
Sprain	19 (12.7%)	13 (8.7%)	14 (9.3%)	13 (8.7%)
Dental pain	17 (11.3%)	13 (8.7%)	14 (9.3%)	14 (9.3%)
Tendonitis	6 (4.0%)	11 (7.3%)	4 (2.7%)	10 (6.7%)
Others (fracture, bursitis, migraine, blunt trauma, tenosynovitis, cervical pain, spondylosis)	20 (13.3%)	28 (18.7%)	26 (17.3%)	25 (16.7%)

Efficacy

**Figure 2:**
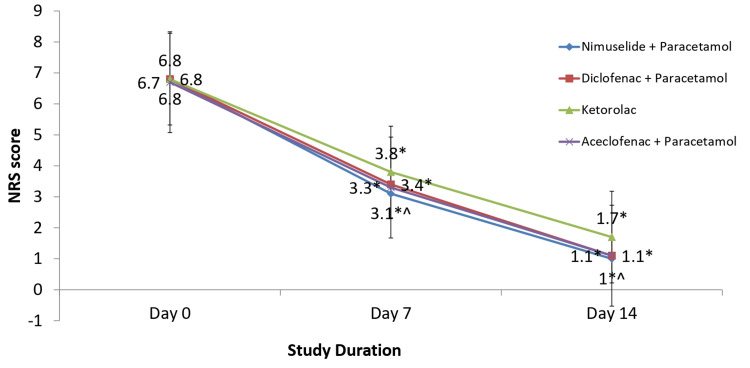
Mean comparison of numerical rating scale (NRS) scores during the study ^*^Pain reduction on day 14 and day seven was significant compared to baseline in all three groups (P<0.001). ^^^Pain reduction on day 14 and day seven in the nimesulide + paracetamol group was significantly more when compared to the ketorolac group (p<0.001) and similar when compared to the diclofenac + paracetamol group and aceclofenac + paracetamol group N = 150 for all groups at all time points

In the primary endpoint analysis, the reduction of pain as measured by NRS at the end of day seven from baseline was 3.75 ± 1.58 in the NP group, 2.96 ± 1.18 in the Kt group, 3.42 ± 1.42 in the DP group, and 3.47 ± 1.30 in the AP group. The pain reduction in all four groups from baseline to day seven was statistically significant (p<0.001). The difference in pain reduction between the NP group and Kt group was found to be statistically significant (p<0.001); however, it was similar in the NP group, DP group, and AP group [mean difference = 0.31 (-0.06 to 0.68) as compared to DP and mean difference = 0.26 (-0.13 to 0.65) as compared to AP on day seven]. The reduction in pain as measured by NRS at the end of day 14 was 5.81 ± 1.76 in the NP group, 5.07 ± 1.77 in the Kt group, 5.67 ± 1.82 in the DP group, and 5.54 ± 1.57 in the AP group as compared to baseline. This pain reduction in all four groups from baseline to day 14 was also statistically significant (p<0.001). As on day seven, the difference in pain reduction between the NP group and Kt group was found to be statistically significant on day 14 as well (p<0.001), and similar between the NP group, the DP group, and the AP group [mean difference = 0.14 (-0.33 to 0.61) as compared to DP and mean difference = 0.26 (-0.22 to 0.75) as compared to the AP group] (Figure 2). Non-inferiority was concluded between the NP, DP group, and AP group as the lower limit of 95% CI of the difference in the change of pain intensity on both day seven and day 14 was above the predefined margin of -1.0.

Safety

**Figure 3:**
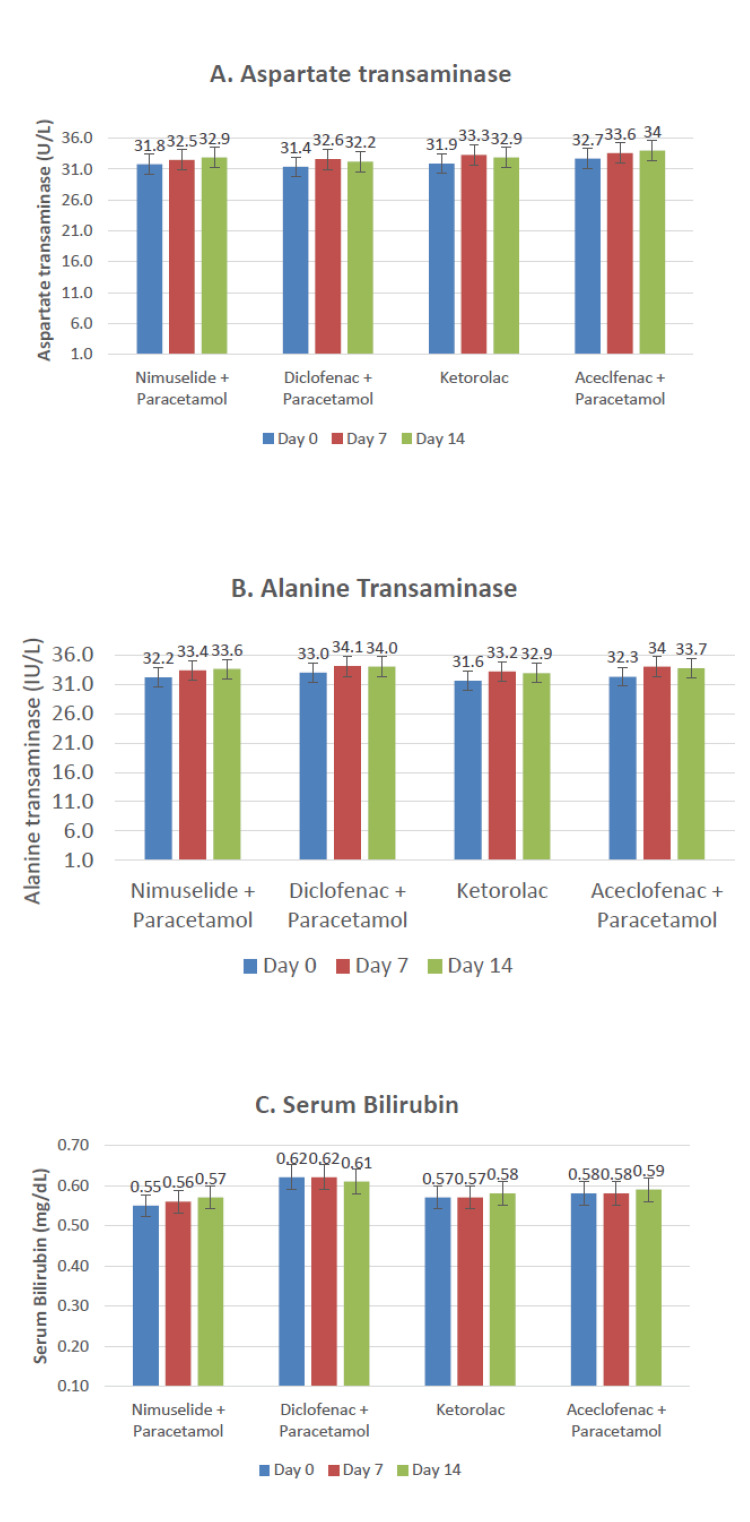
Impact on liver function tests in the four groups during the study There was no significant difference in any parameter on days 7/14 as compared to the baseline in any of the three groups N = 150 for all groups at all time points

In the secondary endpoint analysis, the mean (± SD) difference in liver function tests [ALT/ Serum glutamic pyruvic transaminase (SGPT), AST/serum glutamic-oxaloacetic transaminase (SGOT), and bilirubin] from baseline to day seven and day 14 was neither statistically significant nor clinically significant in the NP group, Kt group, DP group, and AP group. Also, the change in LFT parameters between the NP group, Kt group, DP group, and AP group was statistically non-significant on day seven and day 14 (Figure 3).

**Figure 4:**
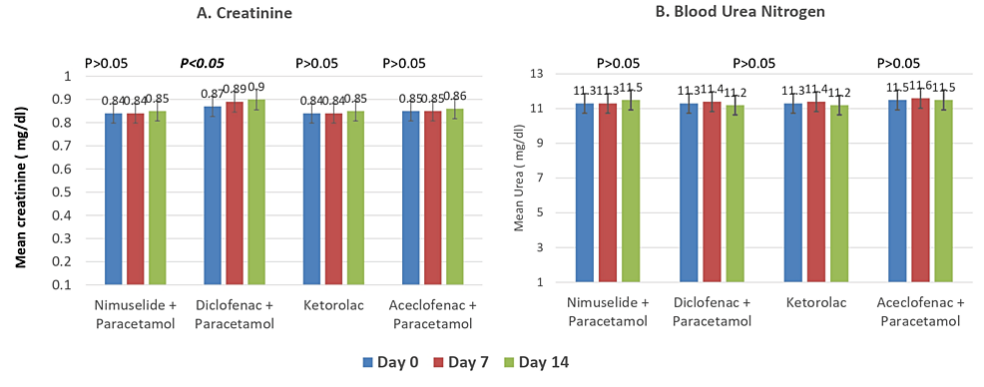
Impact on renal function tests in the three groups during the study N = 150 for all groups at all time points

In another secondary endpoint analysis, the mean (±SD) difference in renal function tests (serum creatinine and BUN) from baseline to day seven and day 14 was not statistically significant in the NP group and Kt group. A statistically significant rise in serum creatinine was noted on day 14 from baseline in the DP group, but the increase in levels was not clinically significant. Also, the difference in serum creatinine between the NP group and DP group was found to be statistically significant on day seven and day 14 (Figure 4); however, the difference was not statistically significant between the NP and Kt groups.

A total of 89 adverse events were reported in the study. Of these 89 TEAEs, 14 were reported in the NP group, 32 in the DP group, 22 in the Kt group, and 21 in the AP group. A significantly greater number of adverse events were reported in the DP group when compared to the NP group (p<0.05). The most common (i.e. ≥1 %) TEAEs reported in the NP group were abdominal pain (3%), elevated liver enzyme (2%), nausea (1.3%), and gastritis (1.3%). The most common (i.e. ≥1 %) TEAEs reported in the DP group were abnormal renal function (4.7%), elevated liver enzyme (4%), and abdominal pain (3.3%); while these were elevated liver enzyme (2.6%), nausea (2.6%), and abdominal pain (2.6%) in the Kt group and abnormal renal function (4.7%), elevated liver enzyme (4%), and abdominal pain (3.3%) in the AP group (Table 2). None of the subjects showed any abnormal ECG changes on day seven and day 14.

**Table 2:** Adverse events reported during the study ^*^P<0.05 as compared to the NP group (Fisher’s exact test) NP: nimesulide (100 mg) + paracetamol (325 mg) tablets; DP: diclofenac (50 mg) + paracetamol (325 mg) tablets; Kt: ketorolac (10 mg); AP: aceclofenac (100 mg) + paracetamol (325 mg) tablets

Adverse events	NP (n = 150), n (%)	DP (n = 150), n (%)	Kt (n = 150), n (%)	AP (n = 150), n (%)
Total	14 (9.3%)	32^*^ (21.3%)	22 (14.7%)	21 (14%)
Nausea	2 (1.3%)	5 (3.3%)	4 (2.6%)	3 (2%)
Vomiting	0 (0.0%)	2 (1.3%)	1 (0.6%)	1 (0.6%)
Gastritis	2 (1.3%)	4 (2.6%)	2 (1.3%)	2 (1.3%)
Abdominal pain	3 (2%)	5 (3.3%)	4 (2.6%)	2 (1.3%)
Dyspepsia	2 (1.3%)	1 (0.6%)	2 (1.3%)	3 (2%)
Vertigo	0 (0.0%)	2 (1.3%)	0 (0.0%)	1 (0.6%)
Diarrhea	1(0.6%)	1 (0.6%)	0 (0.0%)	2 (1.3%)
Headache	0 (0.0%)	1 (0.6%)	2 (1.3%)	0
Abnormal renal function	1 (0.6%)	7 (4.7%)	3 (2%)	3 (2%)
Elevated liver enzymes	3 (2.0%)	4 (4.0%)	4 (2.6%)	5 (3.3%)

## Discussion

This study presents the results of a randomized, prospective, open-label, multicenter, active-controlled study comparing the efficacy and safety of FDC of nimesulide (100 mg) + paracetamol (325 mg) or ketorolac (10 mg) or FDC of diclofenac (50 mg) + paracetamol (325 mg) or aceclofenac (100 mg) + paracetamol (325 mg) in acute painful conditions among Indian patients. NP, DP, and AP were given twice a day while Kt was given three times a day. The combinations were assessed for reduction in pain intensity using NRS scores on day seven and day 14. There was a significant reduction in pain scores on day seven and day 14 from baseline in all four groups. The study revealed that the nimesulide + paracetamol combination is similar in relieving pain to the diclofenac + paracetamol, and aceclofenac + paracetamol, while it is superior to ketorolac alone. The safety profile of NP was similar to that of ketorolac; however, it was significantly better than that of DP and AP (based on renal function test and the number of AEs reported).

FDC of nimesulide + paracetamol has previously been evaluated in Indian patients with acute painful conditions. A multicenter study involving 500 patients by 24 experienced physicians across India showed that the nimesulide + paracetamol combination effectively reduced NRS scores in acute painful conditions and did not lead to clinically significant changes in liver function tests, indicating hepatic safety [13]. A subgroup analysis of the same study also showed that the combination effectively reduced pain and was well-tolerated in all acute painful conditions in a real-world setting without affecting liver functions [14]. This study established the efficacy and safety of the FDC of nimesulide + paracetamol in a real-world setting in India; however, it was a single-arm, non-comparative study. The current study has further established the efficacy and safety of nimesulide + paracetamol in comparison with other commonly used NSAIDs.

Our findings align with those of Shikhkerimov et al.'s study, which assessed the efficacy and safety of nimesulide 200 mg/day in the treatment of acute lower back pain. Pain relief and increased mobility in the lumbar spine were observed on the fifth day of treatment with nimesulide, which indicates its effectiveness in restoring the previous functional status of patients with lower back pain [15]. An observational, multicenter, prospective prescription survey by Levrini et al. has found that nimesulide was the most commonly used drug (68%), followed by diclofenac, ketoprofen, and ibuprofen in patients undergoing dental procedures. It was more effective when compared to other NSAIDs in reducing the pain intensity, delaying the time to maximum pain intensity, and providing complete pain relief on the day of the procedure. Of note, 72.6% of patients who were taking nimesulide had complete pain relief versus 54.7% of those treated with other drugs on day one. The results of this study showed that nimesulide was effective in the treatment of acute postoperative pain induced by dental procedures [16]. These results are similar to our study, where significant improvements in pain were seen in the dental pain group.

Nimesulide has been very effective in controlling pain and inflammation for many years. The safety profile of nimesulide is also favorable with regard to reduced probability of gastrointestinal side effects [17,18]. The pharmacokinetic profile of nimesulide is also good as it is rapidly and completely absorbed. It is widely distributed in the synovial fluid where it remains for a longer duration than blood, which explains its effectiveness in controlling joint pain. Nimesulide is a preferential COX-2 inhibitor due to which it has a lower risk of upper gastrointestinal bleeding. The pharmacokinetic profile of nimesulide is not altered in moderate renal impairment [17].

In the present post-marketing study, the secondary outcomes involved the assessment of patients for changes in LFT parameters including ALT, AST, and serum bilirubin levels. We have observed there was no significant increase in the ALT, AST, and serum bilirubin levels in the participants. A post-marketing surveillance study performed by Chandanwale et al. also concurred that the levels of ALT, AST, and serum bilirubin after treatment with nimesulide remained unaltered, which was comparable with our study results [19]. A study by Warrington et al. found that there was no sign of renal toxicity even at higher doses of nimesulide in 16 subjects, which aligns with our study results [20]. In our study, the level of serum creatinine and BUN was within the normal range after treatment with nimesulide + paracetamol but serum creatinine levels increased after treatment with diclofenac + paracetamol and aceclofenac + paracetamol. This finding is consistent with a previously published case report which stated that diclofenac therapy leads to a rapid rise in serum creatinine value [21].

Prostaglandins have a physiological role in maintaining renal blood flow and glomerular filtration rate (GFR) in normal subjects. Renal blood flow is maintained by the balance between the vasoconstrictor effect of the renin-angiotensin system and the vasodilatory effects of prostaglandins [22]. In vascular-depleted states, prostacyclin (PGI2) affects renal homeostatic mechanisms. PGE2 and PGD2 cause renal blood vessel dilatation along with a decrease in renal vascular resistance, which enhances renal perfusion [23]. Thus, when prostaglandin production is blocked by diclofenac, it may lead to peripheral edema, increased blood pressure, hyperkalemia, and acute renal failure [24]. Diclofenac (150 mg/day) was seen to precipitate acute renal failure in elderly patients and patients with vascular depleted states as this dosage appears to impair the renal blood flow and GFR [25]. It can also alter renal functions through reversible renal ischemia due to its effects on renal prostaglandins [22,23]. Diclofenac increases serum creatinine and urea levels in healthy individuals also but they stay within normal limits [26].

Cardiovascular safety of the four study drugs was also thoroughly assessed in the study by performing ECG at baseline, day seven, and day 14 and also by evaluating other cardiac signs and symptoms. None of the patients in our study had any alteration in ECG. Given their comparative cardiovascular safety, it is important to note that each of these drugs carries specific risks. Diclofenac and aceclofenac have been linked to an increased risk of cardiovascular events, including heart attacks and strokes. The increased risk of thrombotic events due to NSAIDs is because of their COX-2 selectivity and is dependent on dose and duration. Healthcare providers must consider individual patient factors, existing cardiovascular conditions, and the available evidence when choosing among these NSAIDs [27,28].

In our study, the incidence of adverse effects was significantly low among patients in the NP group (14, (p<0.05) as compared to that in the DP group (32) and AP group (21). Nausea, vomiting, abdominal pain, and dyspepsia were reported in all the groups; however, the incidence of all gastrointestinal events was lower in the NP group as compared to the DP, Kt, and AP groups. The low incidence of adverse effects of nimesulide may be attributed to its preferential COX-2 inhibition, which corresponds to a lower potential for gastrointestinal side effects as compared to other drugs [17]. In a clinical study comparing the efficacy and safety of nimesulide and diclofenac in patients with acute shoulder, global tolerability was judged by the investigators to be good/very good in 96.8% of patients in the nimesulide group compared with 72.9% among those given diclofenac [29]. A Chinese study compared the tolerability of nimesulide and diclofenac in osteoarthritis of the knee. The safety profile was in favor of nimesulide with significantly fewer adverse effects in general (13% for nimesulide vs. 29% for diclofenac). Furthermore, there were fewer gastrointestinal adverse events in the nimesulide group than diclofenac group (6.7% vs. 30% of total adverse events, p<0.01) [30]. The results of this study agree with ours as significantly fewer side effects were noted in the NP group (20.6%) as compared to the DP (47%) and AP (21) groups (p<0.05).

Limitations

Although this was a large study, it was open-label due to operational issues and difficulty in manufacturing placebos. This might have had an impact on the assessment of efficacy as this was a subjective parameter, but this does not have an impact on the assessment of safety (based on laboratory investigations), which was one of the important endpoints in the study. During this study, the drugs were prescribed at the physician's discretion. We recommend further studies evaluating the safety of NSAIDs in terms of chronic use with a special focus on renal function tests and liver function tests.

## Conclusions

The results of this randomized, prospective, open-label, multicenter, active-controlled study suggest that the FDC of nimesulide with paracetamol is effective in reducing pain in patients with acute painful conditions and is well tolerated when evaluated on cardiovascular, gastrointestinal, renal, and hepatic parameters. This FDC is superior to ketorolac and non-inferior to the FDC of diclofenac + paracetamol and aceclofenac + paracetamol on efficacy parameters. The tolerability profile of the FDC of nimesulide with paracetamol is similar to that of ketorolac but significantly better than that of the FDC of diclofenac with paracetamol and aceclofenac with paracetamol, especially in terms of renal safety. This FDC of nimesulide with paracetamol can be considered the preferred option for the management of acute painful conditions, especially when baseline laboratory tests are not conducted and in patients with other confounding factors affecting renal function.
